# Providence and Disease

**Published:** 1856-01

**Authors:** 


### PROVIDENCE AND DISEASE.

WE do not believe that Providence has any thing to do with the production of sickness or disease, beyond the institution of certain laws which he has made for the government of the world, any more than that he has an agency in the burning of our finger, if we put it in the fire. We think that very many obituary notices are impious, so far as the agency of the Almighty in removing valuable lives is specially charged. That He mercifully *overrules*, we thankfully admit, but that he changes any organic law, or throws up miraculous barriers to resist the ordinary results of their infringement, we do not believe. Our meaning practically is this: had we gone to Norfolk to help the sick, we should have uttered no prayer for protection against the disease *per se*, we would have looked for no preternatural shield to have been thrown around us, but we would have steadily sought for guidance to live in such a manner as was most wisely calculated to give us strength, vital force to resist, and to throw off the causes of the epidemic; we would have hoped for no favor because of the humanity of our mission, but we would have looked for immunity in proportion as we lived up to the laws of our being. Let no *weak brother* take offence at this doctrine, but take courage when we assure him that our view of the subject is the ground of more heartfelt thankfulness than his, while its rationality is so much the more ennobling. We feel thankful, not that He throws around us an abnormal, or preternatural barrier against disease, but that the laws of our being are so lovingly instituted, that their observance is inevitable of safety, health, and happiness, thus offering the highest premium for the cultivation of our intellect in the study of His ways; this very cultivation happyfying us here, and preparing us for a nearer elevation to Himself when time has passed away.

Norfolk was sickly, because it was unwisely located: more sickly this last summer than usual, because its inhabitants have not had the industry and forethought to remove far enough from them, the accumulating garbage of successive years, and to interpose those contrivances which an elevated science would have indicated, had she been importuned. The elements of disease have been accumulating from year to year by a succession of impressions on the constitution of the inhabitants until the culminating point was reached, and nothing more was wanting to the terrible explosion but the application of the match, which was nothing more than a greater variation than common in the warmth and moisture of the season. Either of two things would have caused an immediate disappearance of the pestilence. Submerging the city and suburbs with a foot deep of running water, or a temperature steadily below seventy degrees of Fahrenheit. The reason for these sentiments are not given now, but we propose doing so in some more inviting form in one of our subsequent numbers. We merely hint enough to set our intelligent readers thinking. We love to make people think; it is only the thoughtful who are of any account in a world like this; it is the thoughtless, the heedless multitude who heap want and calamity and disease on themselves and on too many of those with whom they are brought in frequent association. Now for the bone to pick; untying the Gordian knot.

A heat of ninety degrees will always generate *miasm* in damp and dirty localities. This miasm is the cause of epidemic diseases, but it cannot rise through running water, nor can it exist as such at seventy degrees. The reason that epidemics do not promptly abate on the advent of either of the conditions named is, that at any given time, there are some systems just ripening into disease. The great practical lesson taught by these considerations is, that in times of individual or general sickness, our wisdom consists in industriously searching out, and removing the causes of disease, looking humbly to God for suggestive guidance in these investigations, for strength in the prosecution of our activities, with thankful reliance on the triumphant working of the laws which He has ordained.

